# Global burden of pulmonary sarcoidosis from 1990 to 2021: a comprehensive analysis based on the GBD 2021 study

**DOI:** 10.3389/fmed.2025.1585005

**Published:** 2025-05-16

**Authors:** Jinming Cao, Haiqing Li, Xiaofei Yin, Jianqiong Yang, Lingling Pu, Jing Yang

**Affiliations:** ^1^Department of Radiology, Nanchong Central Hospital/The Second Clinical Medical College of North Sichuan Medical College, Nanchong, China; ^2^Demonstration Center for Experimental Teaching in Biomedicine, Chengdu Medical College, Chengdu, China

**Keywords:** pulmonary nodules, global disease burden, epidemiological trends, disease burden, risk factors, sociodemographic index

## Abstract

**Background and objective:**

Pulmonary sarcoidosis is a chronic respiratory disease with a growing global burden. This study systematically assessed the epidemiological trends, disease burden, and associated factors of pulmonary sarcoidosis in 204 countries from 1990 to 2021 to provide evidence for its prevention and control.

**Methods:**

Utilizing Global Burden of Disease 2021 data, we analyzed the incidence, prevalence, mortality, and disability-adjusted life years (DALYs) of pulmonary sarcoidosis, along with age-standardized rates (ASR), across regions, sexes, and age groups. The association between the disease burden and socio-demographic index (SDI) was also explored.

**Results:**

In 2021, global incidence cases reached 390,000, with 43.07 million prevalence cases, 1.88 million deaths and 40.42 million DALYs. Despite decreases in ASR since 1990, absolute numbers rose. In 2021, Andean Latin America had the highest rates of incidence, prevalence, mortality and DALY, while Eastern Europe had the lowest. Nationally, the burden of the disease in 2021 is particularly significant in countries such as Mauritius and Italy. And disease burden was higher in males and the elderly. In addition, the DALY rate for pulmonary nodular disease showed a non-linear relationship with the SDI, with actual and expected DALY rates differing in most regions and countries.

**Conclusion:**

Pulmonary sarcoidosis poses a growing burden globally, with marked regional, demographic, and socioeconomic disparities. Targeted interventions, including early screening, improved healthcare access, and pollution control, are essential to reduce this burden and promote health equity.

## Introduction

In recent years, with the advancement of diagnostic technology and the heightened health awareness among the population, the detection rate of sarcoidosis has been increasing annually (1). Sarcoidosis is an idiopathic multisystem granulomatous disease that primarily affects the lungs and lymph nodes but can also involve other parts of the body. Notably, approximately 20% of patients may progress to pulmonary fibrosis, and 75% of deaths are related to respiratory causes (2). The clinical presentation of the disease is diverse, with a broad spectrum of symptoms ranging from asymptomatic states to progressive and recurrent disease (3). Common clinical symptoms include dry cough, dyspnea, chest pain, fatigue, and weight loss (4). From an imaging perspective, the typical high-resolution computed tomography (HRCT) features of sarcoidosis are distinctive, characterized by bilaterally symmetric, well-defined enlargement of the hilar and mediastinal lymph nodes, accompanied by lymphangitis along the peribronchovascular interstitial spaces and the interlobular septa in the middle and upper lobes (5), with severe cases presenting with pulmonary fibrosis and honeycomb lung changes (6). Despite the relatively benign clinical course in the majority of sarcoidosis patients, the non-specific clinical manifestations and unclear pathogenesis (7) can lead to missed or misdiagnosis, potentially causing treatment delays that affect patients’ physical function and quality of life, ultimately leading to a worse prognosis (8). Currently, the treatment of sarcoidosis primarily relies on corticosteroids (5) and some emerging therapies, such as immunotherapy (9), which have shown promising efficacy and safety. Although most patients have a favorable prognosis after treatment, some may develop chronic or progressive disease, leading to pulmonary fibrosis and respiratory failure (5). Long-term medication use may also lead to various adverse reactions, such as osteoporosis, infections, diabetes (10, 11). These issues undoubtedly exacerbate the physical and psychological burden on patients, severely affecting their quality of life and long-term survival prognosis.

Exploring the epidemiological characteristics and risk factors of sarcoidosis is crucial for deepening our understanding of the disease, guiding clinical practice, and formulating public health policies. Epidemiological studies have revealed significant geographical, racial, and gender differences in the incidence, prevalence, and mortality of sarcoidosis (5, 12). In recent years, the global trend of sarcoidosis has shown an upward trend. For instance, in the United States, the incidence of sarcoidosis increased from 258.5 to 705.7 per 1,000,000 between 2007 and 2018 (13). The 2019 Global Burden of Disease (GBD) statistics indicate that the incidence, mortality, and disability-adjusted life years (DALYs) of interstitial lung diseases and sarcoidosis have increased globally, particularly among individuals aged 70–79 (14). Studies have also shown that sarcoidosis can occur at any age but is more prevalent in young and middle-aged individuals aged 20–40 (7). Geographically, the incidence of sarcoidosis in Western countries is approximately 10–20 per 100,000 people, whereas the incidence is relatively lower in Asian countries (5). In a case analysis of a hospital’s pulmonary clinic, 61% of 1,429 sarcoidosis patients were male, diagnosed about 3 years younger than females, and with later radiographic staging (15). A Japanese sarcoidosis patient survey found that the average age at diagnosis was 50.1 ± 16.4 years, with 79% being female (16). Reports suggest that due to socioeconomic disparities, sarcoidosis in low-income women tends to be more severe (17). Overall, these studies are mostly concentrated in single countries or regions, lacking a systematic global assessment. Moreover, existing studies often adopt cross-sectional designs, making it difficult to capture the dynamic changes in disease burden. Current research on the global trend of sarcoidosis is still limited, and there is an urgent need for large-scale epidemiological surveys worldwide to comprehensively assess the disease burden of sarcoidosis.

The GBD study is a grand international collaborative project aimed at quantifying the incidence, prevalence, mortality, and disability rates of major diseases and injuries worldwide, providing data support for the formulation of global public health policies (18, 19). By integrating various data sources and employing advanced statistical methods, the project generates and continuously updates disease burden estimates for countries and regions worldwide, providing valuable reference for global health research and practice (18). Leveraging the rich data resources of GBD for epidemiological research allows us to gain a comprehensive view of the global disease burden of sarcoidosis and its influencing factors. Based on this, the present study aims to use GBD data to systematically assess the epidemiological trends, disease burden, and related influencing factors of sarcoidosis in 204 countries and 21 regions worldwide from 1990 to 2021. At the same time, this study will delve into the association between disease burden and sociodemographic indices, in order to provide an epidemiological basis for the prevention and control of sarcoidosis worldwide and to inform the development of targeted intervention strategies.

## Data and methods

### Disease definitions and data sources

The data for this study were sourced from the GBD 2021 database.^1^ In this study, we focused specifically on pulmonary sarcoidosis within the broader GBD 2021 disease category of “Interstitial lung disease and pulmonary sarcoidosis.” It is important to note that the GBD 2021 database uses ICD codes D86-D86.2, D86.9, J84-J84.9 to define this combined disease category. These codes encompass both pulmonary sarcoidosis (D86-D86.2, D86.9) and various interstitial lung diseases (J84-J84.9), and our analysis only employed the D86 code series, while carefully excluding other interstitial lung diseases (J84 codes). We extracted estimates of “incidence,” “prevalence,” “deaths,” and “disability-adjusted life years” (DALYs) for pulmonary sarcoidosis from the GBD 2021, along with their 95% uncertainty intervals (UIs). The “measures” selected included “numbers,” “percentages,” and “rates”; the “locations” encompassed 204 countries, 21 regions, and the global total, summing up to 226 geographical locations; “all GBD age groups” were selected for age; and “male, female, both” were chosen for sex. The study period was set from 1990 to 2021, and all factors provided by GBD 2021 for pulmonary sarcoidosis were included in the risk factor analysis.

### Geographical analysis

To investigate the geographical distribution of the pulmonary sarcoidosis burden, we analyzed GBD 2021 data estimated for 204 countries, 21 regions, and the global total. Age-standardized rates (ASRs) per 100,000 population for incidence, prevalence, deaths, and DALYs in 1990 and 2021 were extracted for all age groups and mapped to their respective geographical areas. And ASR of incidence, prevalence, deaths (also known as mortality), and DALYs are was abbreviated as ASIR, ASPR, ASMR, and ASDR, respectively. A primary world map was created, accompanied by seven detailed subplots focusing on specific regions (Caribbean and Central America, Persian Gulf, Balkans, Southeast Asia, West Africa, Eastern Mediterranean, and Northern Europe) to provide a comprehensive visualization of the global burden of sarcoidosis.

### Sex-specific analysis

To examine sex-specific trends in the pulmonary sarcoidosis burden, GBD data for males and females were analyzed separately. ASRs and the number of cases for incidence, prevalence, deaths, and DALYs per 100,000 population for all age groups in 1990 and 2021 were obtained. The results were stratified and compared by sex and measure.

### Age- and sex-specific analysis of global burden of disease

The focus was on 20 age groups, ranging from 1 to 4 years to over 95 years, stratified by sex. The number of cases (in millions) and rates per 100,000 population for incidence, prevalence, mortality, and DALYs were calculated. Combined bar and line charts were created for each indicator, with bars representing the number of cases and dashed lines representing rates. Error bars and confidence intervals were included to illustrate uncertainty.

### Correlation between sociodemographic index and disease burden

The relationship between the Sociodemographic Index (SDI) and the burden of pulmonary sarcoidosis was investigated. Data from the GBD 2021 study were merged with SDI data for global and regional analysis. Scatter plots of DALYs were generated, plotting ASRs against SDI. LOESS smoothing was applied to visualize trends, and Spearman’s correlation test was conducted to quantify the relationship between SDI and disease burden indicators.

### Statistical analysis

All data processing, statistical analysis, and visualization were conducted using R version 4.0.2, with the following packages: dplyr, tidyr, stringr, and arrow for data manipulation and analysis; ggplot2, ggmap, rgdal, RColorBrewer, patchwork, and ggrepel for data visualization; rgdal for geographical spatial analysis; stats for statistical analysis; and writexl for file output. These packages were utilized for various tasks such as data cleaning, reshaping, statistical computation, geographical mapping, correlation analysis, and the creation of complex visualizations.

## Results

### Global level

In 2021, the global incidence of pulmonary sarcoidosis reached 390,000 (95% UI: 346,000–433,000) new cases (Table 1). The ASIR was 4.54 (95% UI: 4.05–5.04) per 100,000 population, representing a 20.6% (95% UI: 15–26.4%) increase since 1990. Concurrently, the prevalence of pulmonary sarcoidosis increased from 1,887,000 (95% UI: 1,609,000–2,207,000) in 1990 to 4,307,000 (95% UI: 3,803,000–4,899,000) in 2021 (Supplementary Table S1), with the ASPR reaching 50.01 (95% UI: 44.24–56.77) per 100,000 population in 2021, marking an 8.7% (95% UI: 4.1–14%) rise since 1990. Mortality due to pulmonary sarcoidosis rose from 55,000 (95% UI: 45,000–68,000) deaths in 1990 to 188,000 (95% UI: 161,000–212,000) in 2021. The ASMR saw a 50.3% (95% UI: 31.7–74.3%) increase, with the ASMR at 2.28 (95% UI: 1.96–2.56) per 100,000 population in 2021. Additionally, DALYs attributed to pulmonary sarcoidosis increased from 371,500 (95% UI: 306,200–453,700) in 1990 to 476,200 (95% UI: 412,600–531,600) in 2021. The ASDR reached 47.62 (95% UI: 41.26–53.16) per 100,000 population in 2021, with a 28.2% (95% UI: 12.9–50.2%) increase since 1990.

**TABLE 1:** Incidence cases, prevalence cases, deaths, and disability adjusted life years (DALYs) for pulmonary sarcoidosis disease in 2021, and percentage change in age standardized rates (ASRs) per 100,000, by global burden of disease region, from 1990 to 2021.

Location	Incidence (95% UI)			Prevalence (95% UI)			Deaths (95% UI)			DALYs (95% UI)		
	**No, in millions (95% UI)**	**ASR per 100,000 (95% UI)**	**Percentage change in ASRs from 1990 to 2021**	**No, in millions (95% UI)**	**ASR per 100 000 (95% UI)**	**Percentage change in ASRs from 1990 to 2021**	**No, in millions (95% UI)**	**ASR per 100,000 (95% UI)**	**Percentage change in ASRs from 1990 to 2021**	**No, in millions (95% UI)**	**ASR per 100,000 (95% UI)**	**Percentage change in ASRs from 1990 to 2021**
Global	0.39 (0.346–0.433)	4.54 (4.05–5.04)	20.6 (15–26.4)	4.307 (3.800–4.900)	50.01 (44.24–56.77)	8.7 (4.1–14)	0.188 (0.161–0.212)	2.28 (1.96–2.56)	50.3 (31.7–74.3)	4.042 (3.490–4.520)	47.62 (41.26–53.16)	28.2 (12.9–50.2)
High-income Asia Pacific	0.044 (0.0384–0.0496)	11.59 (10.25–13.02)	26.7 (18.3–35.4)	0.642 (0.564–0.732)	151.6 (134.19–172.06)	12.6 (6.7–18.7)	0.026 (0.0218–0.0288)	4.36 (3.73–4.76)	60.3 (43.9–74.8)	0.433 (0.380–0.476)	86.32 (76.64–94.93)	30.3 (18.8–40.7)
High-income North America	0.067 (0.0584–0.0752)	10.95 (9.73–12.2)	29.1 (22.3–36.7)	0.788 (0.695–0.893)	127.5 (113.42–143.85)	9.8 (4.7–16.1)	0.03 (0.0261–0.0315)	4.25 (3.75–4.49)	90.2 (79.9–97.3)	0.583 (0.533–0.622)	90.44 (83.28–96.54)	46.7 (40.2–51.9)
Western Europe	0.044 (0.0396–0.0477)	5.3 (4.82–5.81)	45.4 (40.1–51.7)	0.492 (0.442–0.547)	58.14 (52–65.11)	26.2 (20.5–32.7)	0.031 (0.0268–0.0328)	2.79 (2.49–2.97)	151.4 (133.2–166.7)	0.526 (0.478–0.559)	56.37 (51.97–59.88)	104.9 (92.5–117.1)
Australasia	0.003 (0.00307–0.00372)	6.45 (5.88–7.02)	88 (78.8–98.8)	0.033 (0.0297–0.0365)	61.8 (55.39–68.95)	69 (59.6–80.4)	0.002 (0.0016–0.00209)	3.17 (2.69–3.46)	180.3 (151.7–209)	0.033 (0.029–0.0359)	59.97 (53.13–64.9)	148.7 (126.5–170.1)
Andean Latin America	0.012 (0.0111–0.0125)	20.47 (19.15–21.69)	66.5 (58.5–74.6)	0.08 (0.074–0.0853)	135.98 (126.12–145.58)	77.5 (70.8–84.9)	0.006 (0.00488–0.00802)	11.37 (8.69–14.33)	44.7 (−4.6–122)	0.122 (0.0966–0.150)	209.34 (165.81–257.66)	32.3 (−9.5–95.2)
Tropical Latin America	0.007 (0.00582–0.00744)	2.62 (2.29–2.94)	7.4 (−1.1–16)	0.053 (0.0456–0.0606)	20.45 (17.76–23.47)	−25.5 (−32.4–−17.4)	0.005 (0.00408–0.00484)	1.83 (1.64–1.96)	86.7 (71.7–102)	0.103 (0.0955–0.108)	40.58 (37.64–42.74)	53.7 (43.2–64.5)
Central Latin America	0.012 (0.0109–0.0134)	4.81 (4.33–5.29)	33.3 (26.5–40.6)	0.116 (0.103–0.130)	45.91 (41.07–51.56)	17.6 (11.4–24)	0.006 (0.00584–0.00713)	2.69 (2.42–2.96)	86 (67.9–106.2)	0.158 (0.144–0.174)	62.84 (57.11–69.1)	75.1 (58.4–94.1)
Southern Latin America	0.008 (0.00791–0.00906)	9.9 (9.26–10.57)	65.4 (59.6–71.1)	0.086 (0.0788–0.0926)	99.07 (91.46–107.24)	66.4 (59.5–73.2)	0.004 (0.00391–0.00468)	4.8 (4.31–5.15)	86 (68.7–104)	0.086 (0.0798–0.0922)	98.58 (91.54–105.26)	63.1 (49.9–76.4)
Caribbean	0.001 (0.000917–0.00109)	1.89 (1.73–2.07)	33.7 (28.5–39.5)	0.011 (0.0099–0.0126)	21.09 (18.65–23.82)	37.2 (29.9–45.7)	0.001 (0.000534–0.000773)	1.19 (0.99–1.45)	70.3 (34.5–112.9)	0.016 (0.0128–0.020)	30 (24.15–38.6)	66 (30.4–110.5)
Central Europe	0.004 (0.00366–0.00444)	2.42 (2.2–2.69)	−6.2 (−8.5–−3.5)	0.07 (0.062–0.0796)	38.39 (33.44–44.05)	0.5 (−2.4–3.9)	0.002 (0.00197–0.00237)	0.97 (0.88–1.06)	−6.7 (−15.9–3.8)	0.053 (0.0485–0.0582)	26.9 (24.41–29.45)	−12.9 (−20.7–−4.1)
Eastern Europe	0.003 (0.0023–0.0031)	1.04 (0.89–1.21)	−49.8 (−52.3–−47.3)	0.053 (0.0437–0.063)	17.88 (14.66–21.42)	−46.5 (−49.4–−43.1)	0.001 (0.00105–0.00126)	0.34 (0.31–0.37)	−69.6 (−72.2–−66.8)	0.034 (0.0312–0.038)	10.78 (9.78–12)	−63.3 (−66.6–−59.7)
Central Asia	0.003 (0.00262–0.00309)	3.38 (3.12–3.65)	0.6 (−2.6–4.5)	0.031 (0.0279–0.0354)	35.86 (32.32–40.28)	3.4 (0.2–6.3)	0.001 (0.000515–0.000779)	0.87 (0.71–1.06)	−47.2 (−59–−31.5)	0.02 (0.0163–0.0245)	23.37 (19.37–28.78)	−42 (−52.8–−26.3)
North Africa and Middle East	0.015 (0.0136–0.0168)	2.85 (2.58–3.15)	30.3 (24.5–37.3)	0.186 (0.161–0.214)	35.72 (31.4–40.69)	45.1 (38.4–54.7)	0.003 (0.00225–0.00453)	0.76 (0.56–1.15)	19.2 (−11.8–81.8)	0.098 (0.0757–0.134)	20.11 (15.54–28.24)	24.2 (−6.7–79.6)
South Asia	0.096 (0.0841–0.109)	6.44 (5.65–7.26)	7.2 (3.6–11.7)	0.791 (0.688–0.911)	51.07 (44.55–58.79)	8.1 (4–12.6)	0.055 (0.0359–0.0742)	4.19 (2.81–5.76)	15.9 (−9–73.6)	1.313 (0.891–1.740)	89.8 (61.01–118.92)	9.9 (−14–63.1)
Southeast Asia	0.011 (0.00986–0.0127)	1.62 (1.42–1.81)	18.9 (14.4–24.8)	0.121 (0.104–0.142)	17.4 (15.03–20.22)	31.6 (25.8–39.4)	0.002 (0.000959–0.00377)	0.33 (0.17–0.66)	13 (−11.2–66.2)	0.059 (0.0341–0.109)	8.93 (5.18–16.51)	11.6 (−11.8–49.8)
East Asia	0.05 (0.0429–0.0576)	2.31 (2.02–2.64)	21.9 (14.4–30.7)	0.648 (0.552–0.759)	29.3 (25.22–34.2)	8.3 (0.8–17.1)	0.008 (0.00518–0.0109)	0.41 (0.25–0.54)	−0.3 (−42.8–53.7)	0.234 (0.171–0.301)	11.01 (8.06–14.14)	−11.2 (−42.5–27.8)
Oceania	0 (0.000385–0.000456)	4.2 (3.91–4.51)	13 (8.4–17.6)	0.005 (0.00439–0.00541)	49.16 (44.66–54.3)	15.6 (11.1–20.2)	0 (0.000119–0.000304)	2.3 (1.42–3.85)	6.4 (−32.3–54.5)	0.008 (0.00558–0.0124)	74.63 (50.21–118.43)	7.6 (−28.8–54.8)
Western Sub-Saharan Africa	0.003 (0.00254–0.00349)	1.13 (0.99–1.28)	−17.5 (−20.6−–13.7)	0.032 (0.0261–0.0388)	12.79 (10.7–15.15)	−9.1 (−12.6–−5.4)	0.002 (0.000919–0.00412)	1.39 (0.54–2.41)	−19.6 (−41.5–10.2)	0.07 (0.0298–0.115)	31.33 (13.04–52.94)	−18.9 (−41.6–12.3)
Eastern Sub-Saharan Africa	0.003 (0.00278–0.00375)	1.53 (1.34–1.72)	0.4 (−2.9–4.3)	0.031 (0.0255–0.037)	14.57 (12.34–17.07)	10 (6.7–14.3)	0.001 (0.00053–0.0031)	0.96 (0.33–2.08)	−6.2 (−38.6–38.8)	0.048 (0.0196–0.0932)	23.45 (9.36–46.54)	−5.3 (−39.3–42.9)
Central Sub-Saharan Africa	0.001 (0.0011–0.00146)	1.88 (1.66–2.1)	4.5 (0.7–9.4)	0.012 (0.0105–0.0148)	18.3 (15.77–21.18)	11.2 (6.3–16.5)	0.001 (0.000242–0.00162)	1.47 (0.51–3.92)	3.9 (−31.3–49.8)	0.021 (0.00847–0.0441)	33.84 (13.17–79.52)	3.5 (−31.4–49)
Southern Sub-Saharan Africa	0.003 (0.00224–0.00293)	4.21 (3.69–4.73)	−10 (−12.7–−6.5)	0.026 (0.0222–0.0301)	41.18 (35.6–47.56)	−9 (−11.7–−5.9)	0.001 (0.000677–0.00131)	2 (1.39–2.65)	−4.1 (−34.8–39.8)	0.026 (0.0185–0.0344)	45.01 (31.73–59.13)	−4.5 (−29.2–26)

### Regional level

Regionally, in 2021, the Andean Latin America [20.47 (95% UI: 19.15–21.69)per 100,000 population], high-income Asia Pacific [11.59 (95% UI: 10.25–13.02) per 100,000 population], and high-income North America [10.95 (95% UI: 9.73–12.20) per 100,000 population] had the highest ASIR for pulmonary sarcoidosis, while Eastern Europe [1.04 (95% UI: 0.89–1.21) per 100,000 population], the Caribbean [1.89 (95% UI: 1.73–2.07) per 100,000 population], and Southeast Asia [1.62 (95% UI: 1.42–1.81) per 100,000 population] had the lowest (Table 1). For ASPR, the high-income Asia Pacific [151.6 (95% UI: 134.19–172.06) per 100,000 population], Andean Latin America [135.98 (95% UI: 126.12–145.58) per 100,000 population], and high-income North America [127.5 (95% UI: 113.42–143.85) per 100,000 population] were highest, while Southeast Asia [17.4 (95% UI: 15.03–20.22) per 100,000 population], Eastern Europe [17.88 (95% UI: 14.66–21.42) per 100,000 population], and the Caribbean [21.09 (95% UI: 18.65–23.82) per 100,000 population] were relatively lowest. The ASMR for pulmonary sarcoidosis were highest in Australasia [3.17 (95% UI: 2.69–3.46) per 100,000 population], Andean Latin America [11.37 (95% UI: 8.69–14.33) per 100,000 population], and the high-income Asia Pacific [4.36 (95% UI: 3.73–4.76) per 100,000 population], and lowest in Eastern Europe [0.34 (95% UI: 0.31–0.37) per 100,000 population], Southeast Asia [0.33 (95% UI: 0.17–0.66) per 100,000 population], and North Africa and Middle East [0.76 (95% UI: 0.56–1.15) per 100,000 population]. Moreover, in 2021, the ASDR for pulmonary sarcoidosis were highest in Andean Latin America [209.34 (95% UI: 165.81–257.66) per 100,000 population], South Asia [89.8 (95% UI: 61.01–118.92) per 100,000 population], and high-income North America [90.44 (95% UI: 83.28–96.54) per 100,000 population], and lowest in Southeast Asia [8.93 (95% UI: 5.18–16.51) per 100,000 population], Eastern Europe [10.78 (95% UI: 9.78–12.00) per 100,000 population], and East Asia [11.01 (95% UI: 8.06–14.14) per 100,000 population].

From 1990 to 2021, pulmonary sarcoidosis showed regional disparities. The ASIR was changed significantly, with the largest increases in Australasia [88% (95% UI: 78.8%–98.8%)], Andean Latin America [66.5% (95% UI: 58.5–74.6%)], and Southern Latin America [65.4% (95% UI: 59.6–71.1%)], and the largest decreases in Eastern Europe [−49.8% (95% UI: −52.3– −47.3%)], Western Sub-Saharan Africa [−17.5% (95% UI: −20.6–−13.7%)], and Central Europe [−6.2% (95% UI: −8.5– −3.5%)] (Table 1). During the same period, the ASPR increased most in Andean Latin America [77.5% (95% UI: 70.8–84.9%)], Oceania [69% (95% UI: 59.6–80.4%)], and Southern Latin America [66.4% (95% UI: 59.5–73.2%)], and decreased most in Eastern Europe [−46.5% (95% UI: −49.4– −43.1%)], Tropical Latin America [−25.5% (95% UI: −32.4– −17.4%)], and Western Sub-Saharan Africa [−9.1% (95% UI: −12.6– −5.4%)] (Table 1). Except for Western Europe [151.4% (95% UI: 133.2–166.7%)], Australasia [180.3% (95% UI: 151.7–209%)], and high-income North America [90.2% (95% UI: 79.9–97.3%)], all other regions experienced a decrease in ASMR. The largest decreases were observed in Eastern Europe [−69.6% (95% UI: −72.2– −66.8%)], Central Asia [−47.2% (95% UI: −59– −31.5%)], and Western Sub-Saharan Africa [−19.6% (95% UI: −41.5– −10.2%)]. Additionally, from 1990 to 2021, all regions except Western Europe [104.9% (95% UI: 92.5–117.1%)], Australasia [148.7% (95% UI: 126.5–170.1%)], and high-income North America [46.7% (95% UI: 40.2–51.9%)] saw a decrease in ASDR, with the largest decreases in Eastern Europe [−63.3% (95% UI: −66.6– −59.7%)], Central Asia [−42% (95% UI: −52.8– −26.3%)], and East Asia [−11.2% (95% UI: −42.5– −27.8%)].

### National level

**FIGURE 1:**
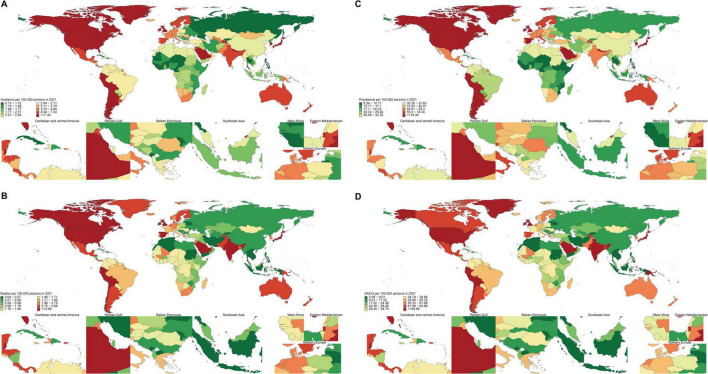
Age-standardized incidence prevalence of pulmonary sarcoidosis, mortality, DALYs per 100,000 population by country, 2021. **(A)** Age standardized point incidence of pulmonary sarcoidosis per 100,000 population in 2021, by country. **(B)** Age standardized point prevalence of pulmonary sarcoidosis per 100,000 population in 2021, by country. **(C)** Age standardized point deaths of pulmonary sarcoidosis per 100,000 population in 2021, by country. **(D)** Age standardized point DALYs of pulmonary sarcoidosis per 100,000 population in 2021, by country.

At the national level, in 2021, the ASIR of pulmonary sarcoidosis ranged from 1.04 to 10.66 per 100,000 population. Mauritius [10.66 (95% UI: 9.91–11.41)], Ireland [8.91 (95% UI: 8.14–9.74)], and Australia [6.61 (95% UI: 6.05–7.19)] had the highest age-standardized incidence rates, while Eastern Europe [1.04 (95% UI: 0.89–1.21)], Turkmenistan [1.27 (95% UI: 1.11–1.45)], and Belarus [1.37 (95% UI: 1.21–1.54)] had the lowest estimates (Figure 1A; Supplementary Table S1). In 2021, the ASPR ranged from 16.69 to 107.87 per 100,000 population. Mauritius [107.87 (95% UI: 98.29–117.39)], Ireland [94.14 (95% UI: 84.70–103.65)], and Ecuador [81.93 (95% UI: 74.99–88.64)] had the highest ASPR, while the Russian Federation [16.69 (95% UI: 13.49–20.22)], Turkmenistan [17.3 (95% UI: 14.33–20.60)], and Eastern Europe [17.88 (95% UI: 14.66–21.42)] had the lowest (Figure 1B; Supplementary Table S2). In 2021, the ASMR ranged from 0.04 to 1.94 per 100,000 population. Italy [1.94 (95% UI: 1.71–2.09)], Saint Vincent and the Grenadines [1.19 (95% UI: 1.04–1.34)], and Libya [0.30 (95% UI: 0.02–1.20)] had the highest ASMR, while the Republic of Moldova [0.04 (95% UI: 0.03–0.05)], Lithuania [0.16 (95% UI: 0.14–0.18)], and Tunisia [0.18 (95% UI: 0.01–0.70)] had the lowest ASMR (Figure 1C;’ Supplementary Table S3). In 2021, the ASDR ranged from 3.06 to 200.52 per 100,000 population. Mauritius [200.52 (95% UI: 184.87–212.76)], Italy [43.50 (95% UI: 39.95–46.84)], and Greece [41.76 (95% UI: 38.48–44.91)] had the highest ASDR, while the Republic of Moldova [3.06 (95% UI: 2.22–4.05)], Lithuania [5.63 (95% UI: 4.66–6.78)], and Belarus [9.01 (95% UI: 7.42–10.69)] had the lowest (Figure 1D; Supplementary Table S4).

From 1990 to 2021, the changes in ASIR of pulmonary sarcoidosis varied widely among countries, with the largest increases in Taiwan, China [113.2% (95% UI: 98.8–127.7%)], Australia [102.9% (95% UI: 93.5–113.2%)], and the Netherlands [98.2% (95% UI: 85.4–110.5%)]. In contrast, Ukraine [−66.5% (95% UI: −68.9– −64.1%)], Belarus [−60.1% (95% UI: −62.6– −57.7%)], and Eastern Europe [−49.8% (95% UI: −52.3– −47.3%)] experienced the largest decreases (Supplementary Table S1). Over the same period, Taiwan, China [118.7% (95% UI: 100.9–140.4%)], Mauritius [113.7% (95% UI: 101.5–124.2%)], and Belize [107.7% (95% UI: 95–121.2%)] had the largest increases in ASPR, while Ukraine [−65.9% (95% UI: −68.9– −62.7%)], Belarus [−52.2% (95% UI: −55.8– −48.6%)], and Eastern Europe [−46.5% (95% UI: −49.4– −43.1%)] had the largest decreases (Supplementary Table S2). Libya [571.9% (95% UI: 267–4809.8%)], Saint Vincent and the Grenadines [537.9% (95% UI: 445.3–642.4%)], and Morocco [455% (95% UI: 309.1–4396.6%)] had the largest increases in ASMR, while the Republic of Moldova [−93.3% (95% UI: −94.2– −92%)], Latvia [−91.9% (95% UI: −93.2– −90.4%)], and Estonia [−89.1% (95% UI: −90.8– −87.1%)] had the largest decreases (Supplementary Table S3). Between 1990 and 2021, Saint Vincent and the Grenadines [458.3% (95% UI: 368.7–562.3%)], Italy [349.5% (95% UI: 289.7–424.8%)], and Greece [289.2% (95% UI: 254.9–321.2%)] had the largest increases in ASDR. In contrast, Latvia [−88.8% (95% UI: −90.9– −86.4%)], Estonia [−84.8% (95% UI: −88– −81.3%)], and Lithuania [−83.2% (95% UI: −86.8– −79.2%)] had the largest decreases (Supplementary Table S4).

### Sex-specific analysis

**FIGURE 2:**
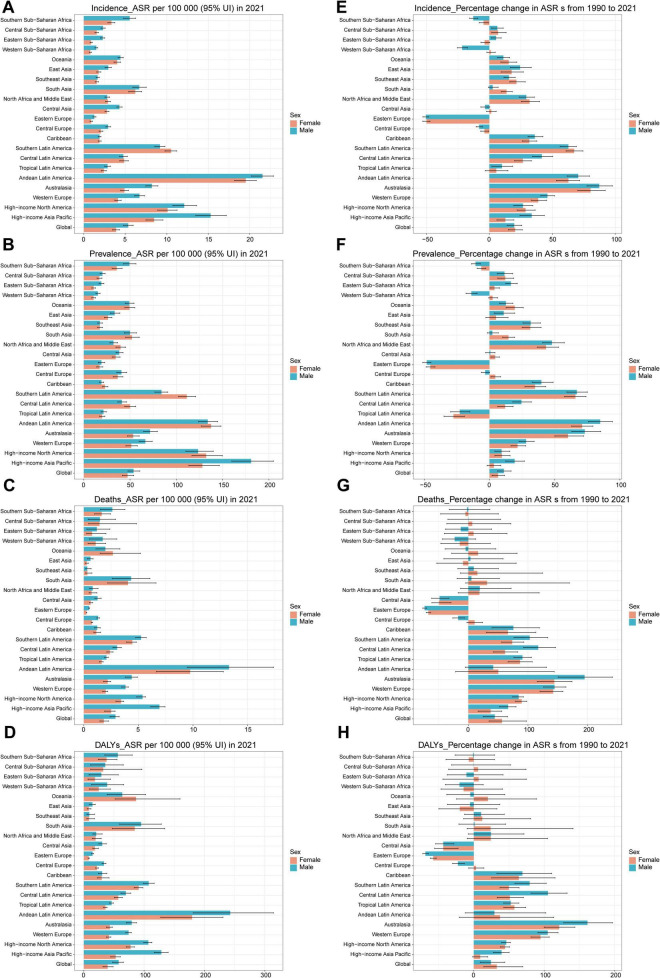
The age-standardized incidence, prevalence, mortality, DALYs in 2021 for the 21 Global Burden of Disease regions, and their percentage change from 1990 to 2021 of pulmonary sarcoidosis disease, by sex. **(A)** The age-standardized point incidence of pulmonary sarcoidosis disease in 2021 for the 21 Global Burden of Disease regions, by sex. **(B)** The age-standardized point prevalence of pulmonary sarcoidosis disease in 2021 for the 21 Global Burden of Disease regions, by sex. **(C)** The age-standardized point deaths of pulmonary sarcoidosis disease in 2021 for the 21 Global Burden of Disease regions, by sex. **(D)** The age-standardized point DALYs of pulmonary sarcoidosis disease in 2021 for the 21 Global Burden of Disease regions, by sex. **(E)** The percentage change in the age-standardized point incidence of pulmonary sarcoidosis from 1990 to 2021 for the 21 Global Burden of Disease regions, by sex. **(F)** The percentage change in the age-standardized point prevalence of pulmonary sarcoidosis from 1990 to 2021 for the 21 Global Burden of Disease regions, by sex. **(G)** The percentage change in the age-standardized point deaths of pulmonary sarcoidosis from 1990 to 2021 for the 21 Global Burden of Disease regions, by sex. **(H)** The percentage change in the age-standardized point DALYs of pulmonary sarcoidosis from 1990 to 2021 for the 21 Global Burden of Disease regions, by sex.

Globally, in 2021, the ASIR for pulmonary sarcoidosis were 5.36 (95% UI: 4.8–5.95) per 100,000 population for males and 3.89 (95% UI: 3.46–4.31) per 100,000 population for females. The highest ASIR for males were observed in the high-income Asia Pacific [15.28 (95% UI: 13.49–17.18)] and for females in the same region [8.47 (95% UI: 7.49–9.55)], while the lowest rates for males were in Eastern Europe [1.27 (95% UI: 1.1–1.47)] and for females in Western Sub-Saharan Africa [0.79 (95% UI: 0.67–0.92)] (Figure 2A). In 2021, the ASPR were 53.73 (95% UI: 47.69–60.59) for males and 47.3 (95% UI: 41.74–54.01) per 100,000 population for females (Figure 2B). The highest ASPR for males were in the high-income Asia Pacific [180.76 (95% UI: 159.93–205.04)] and for females also in the same region [128.17 (95% UI: 113.59–146.48)], while the lowest rates for males were in the Caribbean [19.08 (95% UI: 16.88–21.58)] and for females in Eastern Sub-Saharan Africa [10.64 (95% UI: 8.65–12.92)]. Globally, in 2021, the ASMR for pulmonary sarcoidosis were 2.90 [95% UI: 2.4–3.24)] for males and 1.83 [95% UI: 1.48–2.27] per 100,000 population for females. The highest ASMR for males were in the high-income Asia Pacific [6.92 (95% UI: 6.13–7.44)] and for females in Oceania [2.66 (95% UI: 1.5–5.19)], while the lowest rates for males were in Southeast Asia [0.32 (95% UI: 0.17–0.67)] and for females in Eastern Europe [0.24 (95% UI: 0.22–0.27)] (Figure 2C). In 2021, the ASDR were 57.79 (95% UI: 47.5–65.77) for males and 39.49 (95% UI: 31.95–48.62) per 100,000 population for females. The highest ASDR for males were in Andean Latin America [242.85 (95% UI: 181.45–314.54)] and for females also in the same region [179.56 (95% UI: 127.56–230.82)], while the lowest rates for males were in Southeast Asia [9.18 (95% UI: 5.29–17.89)] and for females in East Asia [8.43 (95% UI: 5.82–11.89)] (Figure 2D]. Overall, globally, males had higher ASIR, ASPR, ASMR, and ASDR than females. There were significant regional disparities in the ASPR, with males and females in the high-income Asia Pacific region having higher rates across incidence, prevalence, and mortality, while those in Andean Latin America had the highest ASDR. In contrast, males and females in Eastern Europe, Southeast Asia, and Sub-Saharan Africa had lower rates across multiple indicators.

From 1990 to 2021, the global ASIR for pulmonary sarcoidosis increased by 19.6% (95% UI: 17.8–23.4%) for males and 20.4% (95% UI: 17.1–23.6%) for females. The largest increases were observed in males and females in Australasia [males: 87.2% (95% UI: 84–89.8%); females: 80.1% (95% UI: 76.7–84.2%)], while the largest decreases were in Eastern Europe [males: −50.6% (95% UI: −50.8– −50%); females: −49.7% (95% UI: −50.7– −49.3%)] (Figure 2E). Over the same period, global ASPR increased by 11% (95% UI: 7.3–15.1%) for males and 6.5% (95% UI: 4–9.4%) for females. The largest increases were observed in males and females in Andean Latin America [males: 84.9% (95% UI: 82.7–87.2%); females: 71% (95% UI: 66.6–74.3%)], while the largest decreases were in Eastern Europe [males: −48.1% (95% UI: −49.3– −48%); females: −45.4% (95% UI: −45.8– −45.1%)] (Figure 2F). Between 1990 and 2021, global ASMR increased by 44.3% (95% UI: 30.6–49.1%) for males and 56.4% (95% UI: 41.9–64.4%) for females. The largest increases were observed in males and females in Australasia [males: 194.6% (95% UI: 188.6–199.4%); females: 143.3% (95% UI: 129.1–151%)], while the largest decreases were in Eastern Europe [males: −73.5% (95% UI: −74.1– −72.6%); females: −67.1% (95% UI: −67.6– −65.4%)] (Figure 2G). From 1990 to 2021, global ASDR increased by 24.3% (95% UI: 14.2–29.7%) for males and 32.6% (95% UI: 21–37.5%) for females. The largest increases were observed in males and females in Australasia [males: 161.4% (95% UI: 160.1–162.1%); females: 121.2% (95% UI: 112.3–121.4%)], while the largest decreases were in Eastern Europe [males: −68.7% (95% UI: −69– −68.1%); females: −57.6% (95% UI: −59.7– −55.4%)] (Figure 2H). Overall, between 1990 and 2021, most regions globally experienced varying degrees of increase in ASIR, ASPR, ASMR, and ASDR for sarcoidosis, reflecting a trend of increasing disease burden. Notably, Australasia and Andean Latin America had significant increases, indicating a more severe situation for pulmonary sarcoidosis control in these regions.

### Age and sex patterns

**FIGURE 3:**
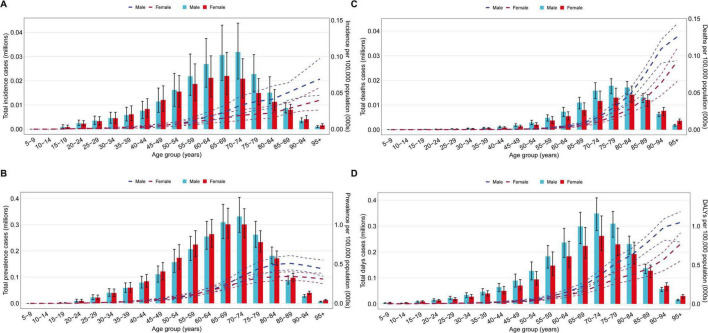
The incidence, prevalence, mortality and DALYs of global pulmonary sarcoidosis in 2021, by age and sex. Lines indicate prevalent case with 95% uncertainty intervals for men and women. **(A)** Number of incidence cases globally and prevalence of pulmonary sarcoidosis disease per 100,000 population, by age and sex in 2021. **(B)** Number of prevalent cases globally and prevalence of pulmonary sarcoidosis disease per 100,000 population, by age and sex in 2021. **(C)** Number of death cases globally and prevalence of pulmonary sarcoidosis disease per 100,000 population, by age and sex in 2021. **(D)** Number of DALYs cases globally and prevalence of pulmonary sarcoidosis disease per 100,000 population, by age and sex in 2021.

From the perspective of the number of incidence, the global number of new cases of pulmonary sarcoidosis peaked in the 70–74 years age group, then decreased with increasing age. Among those under 74 years of age, the number of new cases in males was higher than in females, but among those over 74 years of age, the number of new cases in females exceeded that in males. For example, in the 70–74 years age group, the number of new cases in males was 318,800 (95% UI: 207,300–438,000), higher than that in females at 208,300 (95% UI: 128,500–290,900); whereas in the 80–84 years age group, the number of new cases in females was 113,000 (95% UI: 77,400–163,600), higher than that in males at 150,700 (95% UI: 104,800–216,700). In 2021, the global incidence of pulmonary sarcoidosis began to increase in the 15–19 years age group, peaking in the 95 + age group. Both males and females showed similar trends, with some differences observed across age groups. For instance, in the 15–19 years age group, the ASIR for males was 8.67 (95% UI: 3.39–17.06) per 100,000 population, slightly higher than that for females at 8.32 (95% UI: 3.18–16.50) per 100,000 population; whereas in the 95 + age group, the incidence rate for females was 11.97 (95% UI: 7.93–17.22) per 100,000 population, lower than that for males at 20.70 (95% UI: 13.98–29.24) per 100,000 population (Figure 3A).

Overall, the global number of prevalent cases and ASPR of pulmonary sarcoidosis increased with age but showed a decreasing trend in the elderly population. At the same time, significant sex differences were observed, with higher prevalence and number of prevalent cases in males than in females among middle-aged and younger individuals, while the opposite was observed in the elderly population. Specifically, the global number of prevalent cases of pulmonary sarcoidosis peaked in the 70–74 years age group, then decreased with increasing age. Among those under 74 years of age, the number of prevalent cases in males was higher than in females, but among those over 74 years of age, the number of prevalent cases in females exceeded that in males. For example, in the 70–74 years age group, the number of prevalent cases in males was 331,600 (95% UI: 271,200–404,500), higher than that in females at 301,200 (95% UI: 250,400–361,200); whereas in the 80–84 years age group, the number of prevalent cases in females was 171,600 (95% UI: 146,200–200,000), higher than that in males at 181,400 (95% UI: 151,100–214,900) (Figure 3B). In 2021, the global ASPR began to increase in the 20–24 years age group, peaking in the 95 + age group. Both males and females showed similar trends, with some differences observed across age groups. For instance, in the 20–24 years age group, the ASPR for males was 950.50 (95% UI: 523.60–1,582.60) per 100,000 population, slightly higher than that for females at 922.70 (95% UI: 509.30–1,555.60) per 100,000 population; whereas in the 95 + age group, the ASPR for males was 13,144.6 (95% UI: 10,336.80–16,486.02) per 100,000 population, higher than that for females at 9,281.63 (95% UI: 7,502.4–11,553.29) per 100,000 population.

Regarding the number of deaths, both males and females reached their highest number of deaths in the 80–84 years age group, then decreased with increasing age. Among those under 85–89 years of age, the number of deaths due to pulmonary sarcoidosis in males was higher than in females. For example, in the 80–84 years age group, the number of deaths in males was 17,100 (95% UI: 14,100–19,600), higher than that in females at 14,100 (95% UI: 11,100–17,700); whereas in the 85–89 years age group, the number of deaths in males was 13,100 (95% UI: 11,000–14,600), still higher than that in females at 12,100 (95% UI: 9,300–14,200). In 2021, the global ASMR of pulmonary sarcoidosis reached its highest level in the 95 + age group, with males consistently having higher rates than females across all age groups. For instance, in the 95 + age group, the ASMR for males was 376.8 (95% UI: 279.5–429.7) per 100,000 population, higher than that for females at 277.0 (95% UI: 195.7–330.3) per 100,000 population. In the 5–9 years age group, the ASMR for males was 33.1 (95% UI: 22.30–42.40) per 1,000,000 population, also higher than that for females at 48.5 (95% UI: 31.30–62.90) per 1,000,000 population (Figure 3C). Overall, the global mortality rate of pulmonary sarcoidosis increased with age, reaching its highest level in the elderly population, with males consistently having higher rates than females.

Regarding the number of DALYs, the global number of DALYs due to pulmonary sarcoidosis peaked in the 70–74 years age group, and among those under 85–89 years of age, the number of DALYs in males was higher than in females. For example, in the 70–74 years age group, the number of DALYs in males was 348,600 (95% UI: 282,600–409,000), higher than that in females at 262,200 (95% UI: 205,700–339,300); whereas in the 85–89 years age group, the number of DALYs in males was 137,600 (95% UI: 117,200–153,100), still higher than that in females at 128,100 (95% UI: 101,200–149,800). In 2021, the global ASDR for males with pulmonary sarcoidosis increased with age, peaking in the 85–89 years age group, then decreased with further increases in age. In contrast, the ASDR for females continued to increase up to the 95 + years age group. In all age groups, the ASDR for males was higher than for females. For instance, in the 85–89 years age group, the ASDR for males was 2,392.5 (95% UI: 2,038.7–2,661.9 per 100,000 population), higher than that for females at 1,349.6 (95% UI: 1,066.1–1,579.0) per 100,000 population; whereas in the 95 + years age group, the ASDR for males was 3,141.5 (95% UI: 2,385.7–3,558.9) per 100,000 population, still higher than that for females at 2,309.7 (95% UI: 1,658.2–2,739.6) per 100,000 population (Figure 3D).

### Association with sociodemographic index

**FIGURE 4:**
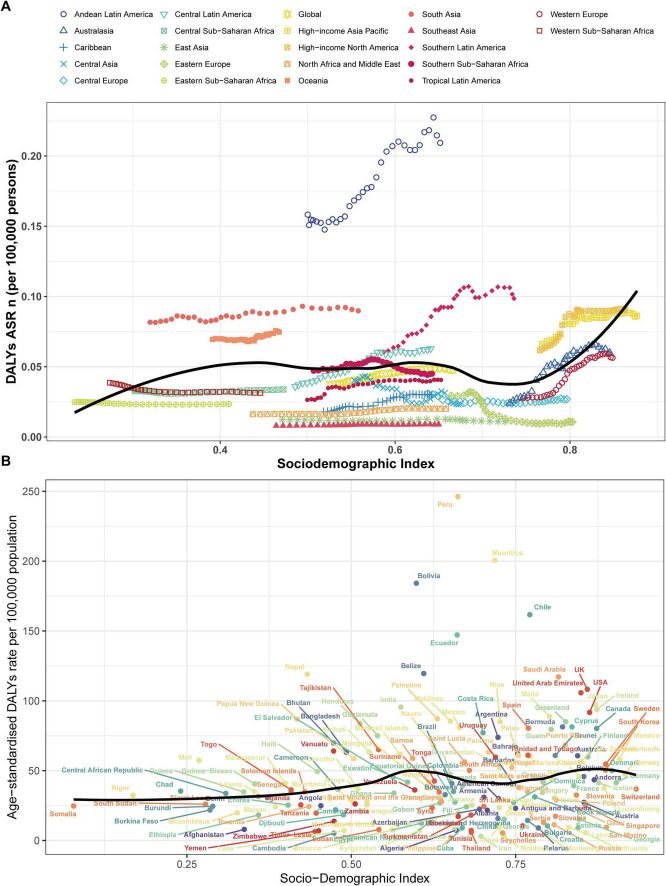
Age-standardized DALY rates for pulmonary sarcoidosis in 21 regions and 204 countries by socio-demographic index, 1990–2021. **(A)** Age-standardized DALY rates for pulmonary sarcoidosis in 21 regions by socio-demographic index. Thirty points were plotted for each region showing the age-standardized DALY rate observed in that region from 1990 to 2021. Expected values for sociodemographic indices and disease rates based on all locations are shown as solid lines. **(B)** Age-standardized DALY rates for pulmonary sarcoidosis in 204 countries by socio-demographic index. Expected values based on the Socio-demographic Index and disease rates in all locations are shown as the black line. Each point shows the observed age-standardized DALY rate for each country in 2021.

At the regional level, we found a correlation coefficient (R) of 0.14 (95% UI: 0.06–0.21) (*P* < 0.001) between the ASDR of pulmonary sarcoidosis and the SDI. However, the association pattern was unique. TheASDR initially increased exponentially with increasing SDI, reaching the first peak when the SDI reached about 0.4, then remained relatively stable until the SDI reached around 0.75, after which it rose again (Figure 4A). Between 1990 and 2021, regions such as Andean Latin America, South Asia, Oceania, and high-income North America had higher ASDR than expected based on their SDI. In contrast, countries in Southeast Asia, East Asia, North Africa, and the Middle East had significantly lower disease burdens of pulmonary sarcoidosis than expected. At the national level, a non-linear association pattern was also observed between the ASDR of pulmonary sarcoidosis and the SDI. The ASDR initially increased with the SDI, reaching a peak when the SDI reached about 0.55, then began to decrease until the SDI reached around 0.75. Subsequently, the ASDR rose again, reaching another peak when the SDI reached 0.85, after which it began to decrease again (Figure 4B). In 2021, countries such as Peru, Mauritius, Bolivia, Chile, and Ecuador had disease burdens significantly higher than expected based on their SDI. In contrast, countries such as Cambodia, Morocco, Thailand, Iran, and Croatia had disease burdens significantly lower than expected. These results indicate that the disease burden of pulmonary sarcoidosis has a complex non-linear association with the level of socio-economic development, and the differences in disease burden among countries may be influenced by various factors.

## Discussion

### Key findings

This study leverages the latest data from the GBD 2021 on the prevalence, mortality, and DALYs of pulmonary sarcoidosis from 1990 to 2021 across 21 regions and 204 countries worldwide, along with their ASRs. Globally, in 2021, there were 4.307 million prevalent cases, 188,000 deaths, and 4.042 million DALYs attributed to pulmonary sarcoidosis. Although the ASPR, ASMR, ASDR have declined over the past three decades, the absolute numbers have increased, underscoring the formidable challenge faced in global public health. The epidemiological characteristics of pulmonary sarcoidosis exhibit significant differences across genders and age groups, particularly among the elderly population, where prevalence, mortality, and DALYs are notably higher. This highlights the need for more targeted prevention and control measures for the elderly. Furthermore, the study revealed a unique non-linear association between the SDI level and ASDR, a finding that not only enriches our understanding of the socioeconomic factors influencing sarcoidosis but also provides crucial support for developing differentiated and effective prevention and control strategies.

### Comparison with other studies

According to the GBD 2017 data, the global prevalence of interstitial lung disease (ILD) and sarcoidosis reached 0.0816% (95% UI: 0.0741–0.0896) in 2017, with an age-standardized DALY rate of 44.04 per 100,000 population (20). The GBD 2019 report further indicated that the global incidence and mortality of these two diseases reached 24.2 million cases and 169,833 deaths, respectively (14). From 1990 to 2019, the global incidence, mortality, and DALYs caused by these diseases increased by 118.6, 166.63, and 122.87%, respectively, revealing their significant impact on global public health. Our study delves into the epidemiological trends of pulmonary sarcoidosis from 1990 to 2021. During this period, the ASIR, ASPR, ASMR, and ASDR of pulmonary sarcoidosis worldwide increased by 20.6, 8.7, 50.3, and 28.2%, respectively, although the magnitude of these growth rates has slowed when considering absolute numbers. These discrepancies may be attributed to the diversity in data set coverage, temporal scope, and research methodologies across different studies. Furthermore, GBD2021 used updated disease definitions and a more rigorous coding strategy compared to GBD 2019, for example, and these methodological changes may complicate direct comparisons of disease data. Notably, our study is the first to analyze pulmonary sarcoidosis separately, diverging from previous reports that grouped ILD and sarcoidosis together. In contrast, the that grouped ILD and sarcoidosis of dpared arch sto an overestimation of the true burden of pulmonary nodular disease and masked trends in pulmonary nodular disease specificity. Our study was also conducted on the harmonized GBD2021 framework, which is still of high reference value, although there are still some differences in absolute data values. In some comparisons, it was found that our results did also show some differences compared with other pulmonary nodular disease studies. A systematic review study showed that the prevalence of pulmonary sarcoidosis fluctuated widely across studies, ranging from 9 to 80% (21). A long-term follow-up study in Finland from 2002 to 2022 revealed that the annual incidence of sarcoidosis was 17–19 cases per 100,000 people, with the proportion of pulmonary sarcoidosis cases among all granulomatous disease cases decreasing from 62 to 45% (22). Reports from two Dutch hospitals on chest CT scans indicated that the proportion of patients with pulmonary sarcoidosis increased from 38% in 2008 to 50% in 2019 (23). These discrepancies with our findings may stem from differences in study time points, diagnostic criteria, sample sizes, levels of analysis (national versus regional), and methodological differences in assessing the prevalence of sarcoidosis.

### Reasons for changes in the burden of pulmonary sarcoidosis

In recent years, the detection rate of pulmonary sarcoidosis has been on the rise. This trend can be partially attributed to the establishment and implementation of management policies that have alleviated the burden of pulmonary sarcoidosis to a certain extent. The British Thoracic Society (BTS) guidelines (24) and the Fleischner Society guidelines (25) are widely accepted for the management of pulmonary sarcoidosis, offering a comprehensive approach that considers not only the characteristics of the nodules but also the risk factors of individual patients. Moreover, the widespread adoption of lung disease screening, comprehensive efforts to restrict tobacco use, improvements in occupational and environmental safety, reductions in environmental and indoor pollution, increased accessibility to treatments that slow disease progression and alleviate comorbidities, heightened public awareness of preventive strategies, and advancements in diagnostic testing technologies to differentiate pulmonary sarcoidosis from other chronic respiratory diseases have collectively capped the further increase in the burden of sarcoidosis (26, 27). Despite these advancements, many low- and middle-income countries have yet to fully comply with tobacco control policies, which remains a significant challenge (28). Technological advancements, particularly the application of various artificial intelligence algorithms in the prediction of pulmonary sarcoidosis exacerbation, offer the possibility of early prevention and reduced disease risk (29). However, it is important to note that the GBD project uses estimation or modeling processes to calculate disease burden (19), rather than directly reporting actual data. Therefore, there may be a degree of uncertainty in this process, potentially leading to an underestimation of the true burden of pulmonary sarcoidosis and consequently affecting the accuracy of policy decisions.

### Reasons for regional variations in mortality rates

In 2021, Nepal’s ASMR was among the highest globally, reaching 182.5 per 100,000 population, which closely associated with the severe air quality in Nepal. Numerous studies have clearly indicated that environmental exposure has a significant negative impact on respiratory system diseases (30, 31). The burning of tobacco and second-hand smoke, biomass fuels, fossil fuels, and significant pollution from transportation emissions collectively pose a major threat to the pulmonary health of Nepali residents (32, 33). In Nepal, 30.8% of the adult reportedly use tobacco, the average age of initiation is 18.2 years, and 36% of adults and 54.1% of adolescents are exposed to second-hand smoke in the home (34). Surveys have also shown that urban air quality in Nepal has been ranked as one of the worst in the world, with increasing physical infrastructure (e.g., transport vehicles, open burning of plastics and other fuels) being the main reason for the escalating pollution (35). Furthermore, as a low-income country, Nepal faces many socioeconomic challenges. Limited economic resources, high illiteracy rates, lack of understanding of government health care policies, and excessive reliance on traditional treatment techniques may further restrict the behavior of Nepali residents in seeking effective health care (36).

It is noteworthy that when comparing the prevalence and mortality rates of pulmonary sarcoidosis in different regions, we found poor consistency between the two. For example, the high-income North America region has the highest prevalence of pulmonary sarcoidosis, but its mortality rate only ranks eighth. This difference may be attributed to the extensive diagnostic coverage, advanced diagnostic technology, and significant improvement in the management of pulmonary sarcoidosis and related comorbidities in that region (37, 38). In contrast, the Oceania region, despite having the ninth-highest prevalence, has an unusually high mortality rate, which may be related to a higher incidence of premature death among patients. The causes of this situation may include the lack of a pulmonary sarcoidosis deterioration risk prediction system (39), the untimeliness of effective diagnostic tests (40, 41), and poor patient treatment compliance (42). In particular, there is a lack of specificity in the treatment of nodular diseases due to the lack of clarity in their etiology and pathogenesis, and no single biomarker has been identified to detect and monitor the specificity and high sensitivity of the disease (43). These results further highlight the significant differences in the quality of management and care between countries with different income levels. In addition, the main challenges in diagnosing pulmonary sarcoidosis in low- and middle-income countries include incomplete medical services, lack of diagnostic tools, and a lack of well-trained clinical staff capable of accurately performing and interpreting tests (44). In resource-limited areas, it is necessary to ensure that every primary medical institution has sufficient diagnostic equipment to improve early detection rates and increase the chances of successful treatment.

### Reasons for the high burden of pulmonary sarcoidosis in the elderly

The burden of pulmonary sarcoidosis is particularly pronounced in the elderly population, a phenomenon closely related to the normal aging process of the lungs. As age progresses, the lungs undergo a variety of structural and functional changes, including a decline in lung function, tissue remodeling, weakened regenerative capacity, and an increased susceptibility to pulmonary diseases (45). Furthermore, the elderly often experience a decline in health status and the presence of multiple comorbidities (46–48), which exacerbates the severity and mortality of pulmonary sarcoidosis. Studies have indicated that the proportion of individuals aged 75 and above with suspicious malignant pulmonary sarcoidosis and nodules sized 10–20 mm is significantly higher than those under 75 (49). Research also suggests that the elderly are more prone to pulmonary sarcoidosis, with the probability of affected individuals over the age of 66 being more than double that of those aged 45–55 (50). This study also found that the age group of 80–84 years had the highest number of pulmonary-sarcoidosis-related deaths. In addition to physiological factors, environmental factors also play a significant role in the high incidence of pulmonary sarcoidosis in the elderly. Among non-communicable diseases, air pollution contributes notably to lung disease-related deaths (51, 52). Particularly, air pollutants such as PM2.5 and PM10 have been linked to the frailty of the elderly (53). It is estimated that deaths caused by air pollution peak in the 80–84 age group, aligning with the distribution of the burden of pulmonary sarcoidosis and further indicating that the elderly are more susceptible to the adverse effects of air pollution.

### Reasons for gender differences

Similar to previous studies (14), our findings indicate that age-standardized prevalence, mortality, and DALY rates are slightly higher in males than in females, primarily reflecting gender differences in smoking behavior and occupational pollution exposure. However, a deeper exploration of gender differences reveals several noteworthy complex factors. Although smoking is more common among males, some studies speculate that females may face a higher risk of pulmonary sarcoidosis due to smoking. Genetically, there are differences between females and males in their genetic makeup, which may lead to varying susceptibilities to pulmonary sarcoidosis. For instance, sex differences in SNP associations within the HLA genes related to sarcoidosis may lead to different immune responses and inflammatory disease courses, and more females are affected by autoimmune (or chronic inflammatory) diseases compared to their male counterparts (54). This could explain the increased susceptibility to pulmonary sarcoidosis in females under certain conditions. Additionally, sex hormones play a significant role in the formation of granulomas in the context of pulmonary sarcoidosis, and hormone levels predictably change throughout adolescence and adulthood, coinciding with the physiological changes of immunosenescence and advanced age (55). This further emphasizes the importance of gender and age patterns in pulmonary sarcoidosis. Apart from smoking and genetic factors, secondhand smoke exposure is also a significant risk for females. Although females smoke less than males, they are often the primary victims of secondhand smoke exposure, which may increase their risk of lung diseases and other related health conditions (56). In summary, gender differences in pulmonary sarcoidosis are the result of a combination of complex factors, including smoking behavior, occupational pollution exposure, genetic factors, sex hormone levels, and secondhand smoke exposure. When formulating prevention and control strategies, these factors should be taken into full consideration to more effectively reduce the burden of pulmonary sarcoidosis in different genders.

### Main risk factors and prevention measures for pulmonary sarcoidosis

Reports indicate that smoking, environmental particulate matter pollution, and occupational exposure to particles, gases, and fumes are major contributing factors to the burden of pulmonary sarcoidosis (57). Since these risk factors are largely preventable and pulmonary sarcoidosis can be effectively treated, controlling the burden of pulmonary sarcoidosis requires increased attention and concentrated efforts. Several initiatives have been implemented to reduce the risks posed by these factors. Smoking is the most common risk factor for all chronic respiratory diseases (44). A survey in North China found that about 10.09% of the incidence of pulmonary sarcoidosis was attributed to smoking (58). The WHO’s “MPOWER” package includes best-practice, cost-effective intervention measures outlined in the WHO Framework Convention on Tobacco Control (WHO FCTC) to assist countries in reducing tobacco demand (59). By 2020, 136 countries had adopted and maintained at least one highest-level MPOWER policy, which together reduced 81 million smokers and 28.3 million smoking-attributed deaths (60). Interestingly, nicotine, a major component of cigarettes, can modulate a range of inflammations, and smokers chronically exposed to nicotine do not suffer from hyper-sensitive pneumonia and pulmonary sarcoidosis, showing some promise for the treatment of pulmonary sarcoidosis (61, 62). Nevertheless, preventing exposure to tobacco smoke remains an effective long-term strategy for reducing the burden of pulmonary sarcoidosis (63). The United States has made significant progress in controlling air pollution, mainly due to interventions such as the 1990 Clean Air Act Amendment and the 2002 NOx State Implementation Plan requirements, as well as strict regulations on anthropogenic emissions from different types of vehicles (64, 65). These measures may help reduce the mortality burden caused by non-communicable diseases. Exposure to biomass fuel is also a key air pollution factor. Measures such as using clean fuel alternatives, improving kitchen ventilation, and providing better stoves can reduce the risk of pulmonary sarcoidosis and improve patients’ pulmonary dysfunction (66). Therefore, setting strict health measures to prevent smoking and improve air pollution may be a key initiative for healthcare policymakers to alleviate the burden of pulmonary sarcoidosis.

### The relationship between pulmonary sarcoidosis burden and SDI

The relationship between DALYs caused by pulmonary sarcoidosis and the SDI does not follow a linear trend. This pattern of relationship is likely closely related to a variety of socioeconomic factors. In low-income countries, slow socioeconomic development is often accompanied by insufficient health awareness, inadequate social support systems, a lack of healthcare resources, and poor living conditions, as well as widespread use of smoking and biomass fuels (67). Many cases are also not included in the statistics due to inadequate medical conditions. These factors interact with each other, directly or indirectly increasing the risk of pulmonary sarcoidosis. Moreover, smoking, an important risk factor for pulmonary sarcoidosis, is not well controlled in low-income countries. Statistics show that 75% of the reduction in smokers and smoking-attributed deaths from MPOWER policies occurred in middle-income countries, 20% in high-income countries, and less than 5% in low-income countries (60). This further highlights the challenges low-income countries face in controlling risk factors such as smoking. In regions with low-to-middle SDI (around 0.4), the initial peak in ASDR may represent the “epidemiological transition” phenomenon, where countries experience a dual burden of disease. As these economies transition, they face both emerging non-communicable diseases (including sarcoidosis) and persistent infectious diseases, while simultaneously developing but not yet optimizing their healthcare infrastructure. In developing countries with moderate SDI, indoor air pollution caused by the burning of coal and biomass fuels may reflect rapid industrialization and economic growth and is also a major cause of the pulmonary sarcoidosis burden in developing countries (67). But a balance between increasing diagnostic capacity and improvements in healthcare access may offset the true prevalence of the disease. In addition, countries with the largest populations and moderate SDI, such as China, may exhibit a higher disease burden due to the rapid progress of population aging. However, it is important to note that not all low SDI countries face a high burden of pulmonary sarcoidosis. For example, countries in Sub-Saharan Africa, Western and Eastern, and Oceania with lower SDI have lower than expected prevalence and mortality rates of pulmonary sarcoidosis. This may be related to the specific socioeconomic conditions, patterns of healthcare expenditure, or public health policies in these countries. On the contrary, the concomitant rise in ASDR at higher SDI levels may reflect superior diagnostic capacity, stronger monitoring systems, and greater health resources in high-income countries. And even in these countries with higher per capita GDP, their healthcare systems may also have inequities. These countries often have a higher proportion of private healthcare services, which can lead to uneven distribution of health resources and exacerbate health inequities (68, 69).

In general, in areas with limited medical resources, the diagnosis of sarcoidosis is generally low due to the lack of advanced imaging and pathological diagnostic support, and the incidence of sarcoidosis exhibits a significant geographic pattern: differences in the process of industrialization have resulted in very different occupational exposure profiles (e.g., exposure to asbestos, heavy metal contamination) and environmental risk profiles (e.g., air pollution, accumulation of soil toxins), which in turn interacts with genetic susceptibility of the different populations, resulting in a stepwise distribution of incidence. The result is a stepwise distribution of morbidity. This mechanism of bio-environmental interactions is population-specific, further exacerbating geo-epidemiological heterogeneity. Differences in national strategic priorities for health resource allocation magnify the dilemmas of prevention and control—developing countries are constrained by pressures on infectious disease prevention and control to invest in rare disease screening, while developed countries have the technological advantage of dealing with the environmental liabilities inherited from industrialization. Therefore, when formulating prevention and control strategies, it is necessary to fully consider the socioeconomic characteristics, distribution of risk factors, and healthcare systems of different countries to develop more targeted and effective measures to reduce the burden of pulmonary sarcoidosis.

### Study limitations and future prospects

This study utilized data from the GBD to systematically assess the global epidemiological trends and disease burden of pulmonary sarcoidosis but is not without limitations. Firstly, due to variations in disease surveillance and reporting systems across countries and regions, some data may be subject to omissions or quality issues, potentially affecting the accuracy of the results. Secondly, the study primarily focused on the overall trends and disease burden of pulmonary sarcoidosis without analyzing different types of pulmonary sarcoidosis, such as cysts or bullae, which might obscure the heterogeneity of the disease. Whereas the heterogeneity of clinical subtypes associated with pulmonary nodular disease is not adequately discussed in this article and may mask differences in pathomechanisms, prognosis, and response to therapy across phenotypes. Furthermore, while the study explored the relationship between disease burden and socioeconomic development levels, it did not delve into other potential influencing factors, such as healthcare access, environmental exposures, and genetic backgrounds.

Future research should aim to improve disease surveillance and reporting systems to enhance data quality and provide more reliable evidence for global pulmonary sarcoidosis prevention and control. Additionally, more studies targeting different types of pulmonary sarcoidosis are needed to uncover the heterogeneity of the disease. Meanwhile, clinical data should be integrated to establish subtype-specific epidemiological databases to identify different subgroups of the disease and clinically validate the results of targeted therapeutic interventions, which will provide insights for precision prevention and control measures. Moreover, there should be an increased focus on exploring factors that influence disease burden, employing interdisciplinary and multilevel research methods to fully understand the epidemiological patterns and pathogenic mechanisms of pulmonary sarcoidosis. This comprehensive understanding will inform the development of effective prevention and control strategies. Only by continuously deepening our understanding and improving prevention and control systems can we ultimately control the global prevalence of sarcoidosis, reduce the disease burden, and promote human health.

## Conclusion

This study provides a comprehensive analysis of the global disease burden of pulmonary sarcoidosis, revealing its distribution and trends across different regions, countries, age groups, and genders. The findings highlight significant geographical disparities and temporal changes in the disease burden of pulmonary sarcoidosis worldwide. At the regional level, the Andean Latin America, high-income Asia Pacific, and high-income North America regions had the highest incidence and prevalence rates, while Eastern Europe, Southeast Asia, and the Caribbean had the lowest. From 1990 to 2021, age-standardized incidence and prevalence rates of pulmonary sarcoidosis increased in most regions, while age-standardized mortality and DALY rates decreased in the majority of areas. At the national level, there are substantial variations in the disease burden of pulmonary sarcoidosis, reflecting differences in healthcare conditions, environmental factors, and genetic backgrounds among countries. Age and gender analyses indicate that the incidence, prevalence, mortality, and DALY rates of pulmonary sarcoidosis rise with age and are higher in males than in females for most age groups. Furthermore, this study discovered a complex non-linear association between the disease burden of pulmonary sarcoidosis and theSDI, suggesting that the impact of socioeconomic development levels on disease burden is multifaceted. These findings provide crucial insights for the development of targeted prevention and control strategies, which can contribute to improving the management of pulmonary sarcoidosis and patient outcomes worldwide.

## Data Availability

The original contributions presented in this study are included in this article/Supplementary material, further inquiries can be directed to the corresponding author.

## Footnotes

1
https://ghdx.healthdata.org/gbd-2021
